# Oncology and Solid Organ Transplantation: New Biological and Clinical Insights

**DOI:** 10.3389/ti.2026.16528

**Published:** 2026-03-24

**Authors:** Gianluigi Zaza, Annemarie Weissenbacher, Alessandro Vitale, Lorna Marson, Pål-Dag Line, David Cucchiari, Andreas Kronbichler

**Affiliations:** 1 Department of Translational and Precision Medicine, Division of Nephrology, Policlinico Umberto I Hospital, Sapienza University of Rome, Rome, Italy; 2 Department of Visceral, Transplant and Thoracic Surgery, OrganLife Organ Regeneration Center of Excellence, and Daniel Swarovski Research Laboratory, Medical University of Innsbruck, Innsbruck, Austria; 3 Department of Surgical Oncological and Gastroenterological Sciences, Padova University, Padova, Italy; 4 Edinburgh Transplant Centre, Royal Infirmary of Edinburgh, Edinburgh, United Kingdom; 5 Department of Transplantation, Oslo University Hospital, Oslo, Norway; 6 Institute of Clinical Medicine, University of Oslo, Oslo, Norway; 7 Department of Nephrology and Kidney Transplantation, Hospital Clínic of Barcelona, Barcelona, Spain; 8 Department of Internal Medicine IV, Nephrology and Hypertension, Medical University Innsbruck, Innsbruck, Austria; 9 Department of Health, Medicine and Caring Sciences, Linköping University, Linköping, Sweden

**Keywords:** kidney transplantation (KT), liver transplantation (LT), lung transplantation (LTx), oncology, solid organ transplant (SOT)

Solid organ transplantation represents a major challenge in modern medicine, as malignancy is one of the leading comorbidities affecting both patient and graft survival. Cancer risk in this large and heterogeneous population is affected by multiple donor- and recipient-related factors and, most importantly, by long-term immunosuppression [1]. Indeed, immunosuppressive therapies impair immune surveillance, predisposing to oncogenic viral infections and may directly influence tumor behavior [2]. In addition, although rare, donor-transmitted cancer remains a serious concern, requiring stringent assessment protocols and continuously updated guidelines [3].

Significant advances have been made in expanding the donor pool, particularly in liver transplantation, where indications have extended beyond hepatocellular carcinoma to include selected primary and metastatic liver tumors within the evolving field of transplant oncology. At the same time, precision medicine and next-generation sequencing technologies have led to the identification of new diagnostic and therapeutic targets. Immune checkpoint inhibitors are a standard of care for many cancer types, although their use in transplant patients is still challenging.

Therefore, this Special Issue addresses these topics through several rigorous original articles, mainly based on large clinical experiences, and comprehensive reviews. Each contribution addresses a distinct yet interrelated aspect of solid organ transplantation and oncology, collectively providing an in-depth overview of the field’s current achievements and outlining its future transformative perspectives. Briefly, below are reported, in summary, the main evidence and results of the paper included in the special issue titled: *“Oncology and solid organ transplantation: new biological and clinical insights.”*

- In a large 20-year single-center experience with right lateral sector grafts (RLSG) in adult living donor liver transplantation (a 661-case series from the University of Tokyo Hospital), Hayakawa et al. clearly demonstrated the value of this approach in expanding donor options with low donor complication rates and no donor mortality. Recipients experienced higher rates of vascular and biliary complications than those with other graft types; however, 5-year survival was comparable. RLSG increased the donor pool by 5% and represented the only viable option for certain patients. Despite the higher complication rates, it remains a feasible and life-saving alternative when standard grafts are not suitable.
- Pommerolle et al. conducted a multicenter, retrospective, case-control study to evaluate the impact of screening in patients with renal cell carcinoma after kidney transplantation. The patients followed up in centers performing annual screening had smaller cancers, a lower relapse and cancer-related mortality rates, highlighting the importance of implementing annual screening to improve patient prognosis.
- Benoni et al., in a nationwide, population-based study analyzed data from a Swedish national registry to compare 98 organ transplant recipients with colorectal cancer (CRC) to 474 non-transplanted patients and identified differences in cancer characteristics and treatment-related factors that affected patients’ survival. They found that transplant patients with stage I–III CRC were less likely to undergo abdominal surgery than non-transplant patients. Among patients treated with surgery, transplant patients with CRC were less likely to receive adjuvant chemotherapy, and transplant patients with rectal cancer had a higher rate of relapse than non-transplant patients. The 5-year cancer-specific and overall survival rates were lower in transplant patients (56% and 35%, respectively) than in controls (68% and 57%, respectively). The authors suggested that multidisciplinary evaluation involving transplant specialists should be applied in the management of CRC for all organ transplant recipients to ensure an optimal therapeutic strategy for this patient population.
- Stenman et al. studied post-transplant cancer incidence and survival in 664 heart transplant recipients at Sahlgrenska University Hospital. Over a median follow-up of 7.7 years, 19% of patients developed malignancies. The overall cancer risk was 6.2-fold higher than the general population, and 2.9-fold higher when excluding non-melanoma skin cancer (NMSC). The most common cancers were NMSC, non-Hodgkin lymphoma, and lung cancer. The risk factors included age, smoking, hypertension, cytomegalovirus (CMV) status, ischemic time, and azathioprine. A history of prior cancer did not affect post-transplant survival.
- The same research group also highlighted the main factors associated with an increased risk of cancer after lung transplantation. The key determinants were older donor and recipient age, type of immunosuppression, and whether the transplant was single or bilateral. The authors also noted that the improvement in 5-year survival after lung transplantation, from 46% in 1990 to 57% in 2015, should be considered, as longer post-transplant survival may contribute to a higher cumulative risk of cancer Stenman et al.
- Re Sartò et al. investigated the epidemiology of cancer after kidney transplantation, its risk factors, and its impact on management and survival in a retrospective single-center study. An Italian cohort of 930 kidney transplant recipients (KTR) followed up for 7 years was analyzed. During the follow-up, 19% of patients developed cancer, most commonly NMSC (55%). The identified risk factors included older age at the time of transplantation, higher body mass index (BMI) 1 month after transplantation, vasculitis, autosomal dominant polycystic kidney disease (ADPKD), and anti-thymocyte globulin (ATG) induction. ATG was associated with earlier cancer onset, while modification of immunosuppression after cancer diagnosis, particularly conversion to mTOR inhibitors, was linked to improved survival.
- Similarly, Srisuwarn et al. analyzed a Thai single-center cohort of 2,024 kidney transplant recipients and reported that 6.2% developed 133 post-transplant malignancies (over 16,495 person-years at risk). Urothelial cancers and non-Hodgkin lymphoma showed the highest excess risk, with urothelial cancer particularly increased among women. Consistent with the Italian cohort, skin cancers were also among the most frequent malignancies.
- Given the high risk of skin cancers among solid organ transplant recipients and the lack of standardized triage guidelines to assist dermatology clinics in scheduling new patients pre- or post-transplant, Hirotsu et al. developed triage recommendations based on expert panel consensus for dermatologic management of this high-risk population. The proposed algorithm offers practical, risk-stratified guidance to support prevention and prompt diagnosis to reduce the skin cancer burden and progression of high-risk skin cancers in solid organ transplant recipients, together with a reduction of overall healthcare costs.
- Rare malignancies pose a significant challenge in transplantation. Lefevre et al. reported a metastatic donor-derived BKV-induced Bellini duct carcinoma in a kidney transplant recipient, successfully managed without chemotherapy or immunotherapy. Withdrawal of immunosuppression enabled spontaneous tumor rejection through alloimmune and antitumor responses.

Collectively, these studies highlight the importance of pre- and post-transplant cancer surveillance together with the tailoring of maintenance immunosuppressive therapy to address the high cancer risk in this patient population and optimize long-term graft and patient outcomes.

- Vanlerberghe et al. examined *de novo* malignancy (DNM) risk post-liver transplant in a retrospective analysis of 174 patients transplanted for alcoholic liver disease (ALD). Nineteen patients (10.9%) developed DNM between 12 and 60 months post-transplant. Independent risk factors included higher tacrolimus drug exposure, together with other factors (such as older age, greater smoking history, and active smoking at transplant). Minimizing tacrolimus exposure in the first year and promoting smoking cessation before transplantation may reduce the risk of DNM.
- Moreover, as reported by Zakrocka et al., paraneoplastic glomerular diseases, complex renal manifestations associated with malignancy, are currently understudied in transplant recipients but deserve careful evaluation and consideration to ensure long-term preservation of graft function. Ongoing advances in clinical and molecular nephrology are expected to enhance our understanding of the underlying mechanisms and support the development of more effective diagnostic and therapeutic strategies for these conditions.
- Another important topic that has been analyzed in this special issue by Barbir et al. is the use of immune checkpoint inhibitors (ICIs) in KTRs, currently challenged by the risk of allograft rejection and loss. However, analyzing the available literature, the authors concluded that this risk can be reduced by optimizing maintenance immunosuppressive therapy and potentially by close follow-up to enable early intervention. Extra-renal immune-related adverse events are less common, though data on recurrent glomerulonephritis in the allograft are limited. Risk stratification using protocol biopsies, non-invasive biomarkers, or PD-L1 staining could help guide therapy, but further validation is needed. A patient-centered, multidisciplinary approach and unified transplant-oncology protocols are essential, and future studies should compare immunosuppressive strategies to optimize both cancer control and allograft survival.
- This was in line with Bolufer et al. who emphasized that cancer management in KTRs requires a multidisciplinary approach. The use of ICIs demands careful risk-benefit assessment, maintaining at least two immunosuppressants, adjusting corticosteroid dose, and switching from calcineurin inhibitors to mTOR inhibitors. However, further prospective studies are needed to refine these strategies.
- Finally, Turra et al. critically analyzed the current knowledge on the use of donors with known malignancy histories (focusing on cancer types, stages, and patient survival outcomes) to improve strategies for safe and effective organ transplantation from these donors. Individualized and multidisciplinary clinical judgment is essential when using organs from donors with cancer, with recipients being fully informed of risks, including the consequences of not proceeding with transplantation, before giving consent.

In conclusion, this Special Issue, involving clinicians and researchers worldwide, offers a comprehensive overview of this important topic in transplant medicine, providing new clinical evidence, guidelines, and translational research insights. It highlights the importance of innovative multidisciplinary management strategies to improve both allograft and patient outcomes.
